# Ultrasound-induced protein restructuring and ordered aggregation to form amyloid crystals

**DOI:** 10.1007/s00249-022-01601-4

**Published:** 2022-05-16

**Authors:** Rachana Pathak, Sukhvir Kaur Bhangu, Gregory J. O. Martin, Frances Separovic, Muthupandian Ashokkumar

**Affiliations:** 1 School of Chemistry, The University of Melbourne, Melbourne, VIC 3010 Australia; 2 Department of Chemical Engineering, The University of Melbourne, Melbourne, VIC 3010 Australia; 3 The ARC Dairy Innovation Hub, The University of Melbourne, Melbourne, VIC 3010 Australia; 4 Bio21 Institute, The University of Melbourne, Melbourne, VIC 3010 Australia; 5 School of Science, RMIT University, Melbourne, VIC 3000 Australia

**Keywords:** Amyloid, Protein aggregation, β-Lactoglobulin, Amyloid crystals, Ultrasound

## Abstract

Amyloid crystals, a form of ordered protein aggregates documented relatively recently, have not been studied as extensively as amyloid fibres. This study investigates the formation of amyloid crystals with low frequency ultrasound (20 kHz) using β-lactoglobulin, as a model protein for amyloid synthesis. Acoustic cavitation generates localised zones of intense shear, with extreme heat and pressure that could potentially drive the formation of amyloid structures at ambient bulk fluid temperatures (20 ± 1 °C). Thioflavin T fluorescence and electron microscopy showed that low-frequency ultrasound at 20 W/cm^3^ input power induced β-stacking to produce amyloid crystals in the mesoscopic size range, with a mean length of approximately 22 µm. FTIR spectroscopy indicated a shift towards increased intermolecular antiparallel β-sheet content. An increase in sonication time (0–60 min) and input power (4–24 W/cm^3^) increased the mean crystal length, but this increase was not linearly proportional to sonication time and input power due to the delayed onset of crystal growth. We propose that acoustic cavitation causes protein unfolding and aggregation and imparts energy to aggregates to cross the torsion barrier, to achieve their lowest energy state as amyloid crystals. The study contributes to a further understanding of protein chemistry relating to the energy landscape of folding and aggregation. Ultrasound presents opportunities for practical applications of amyloid structures, presenting a more adaptable and scalable approach for synthesis.

**Graphical abstract:**

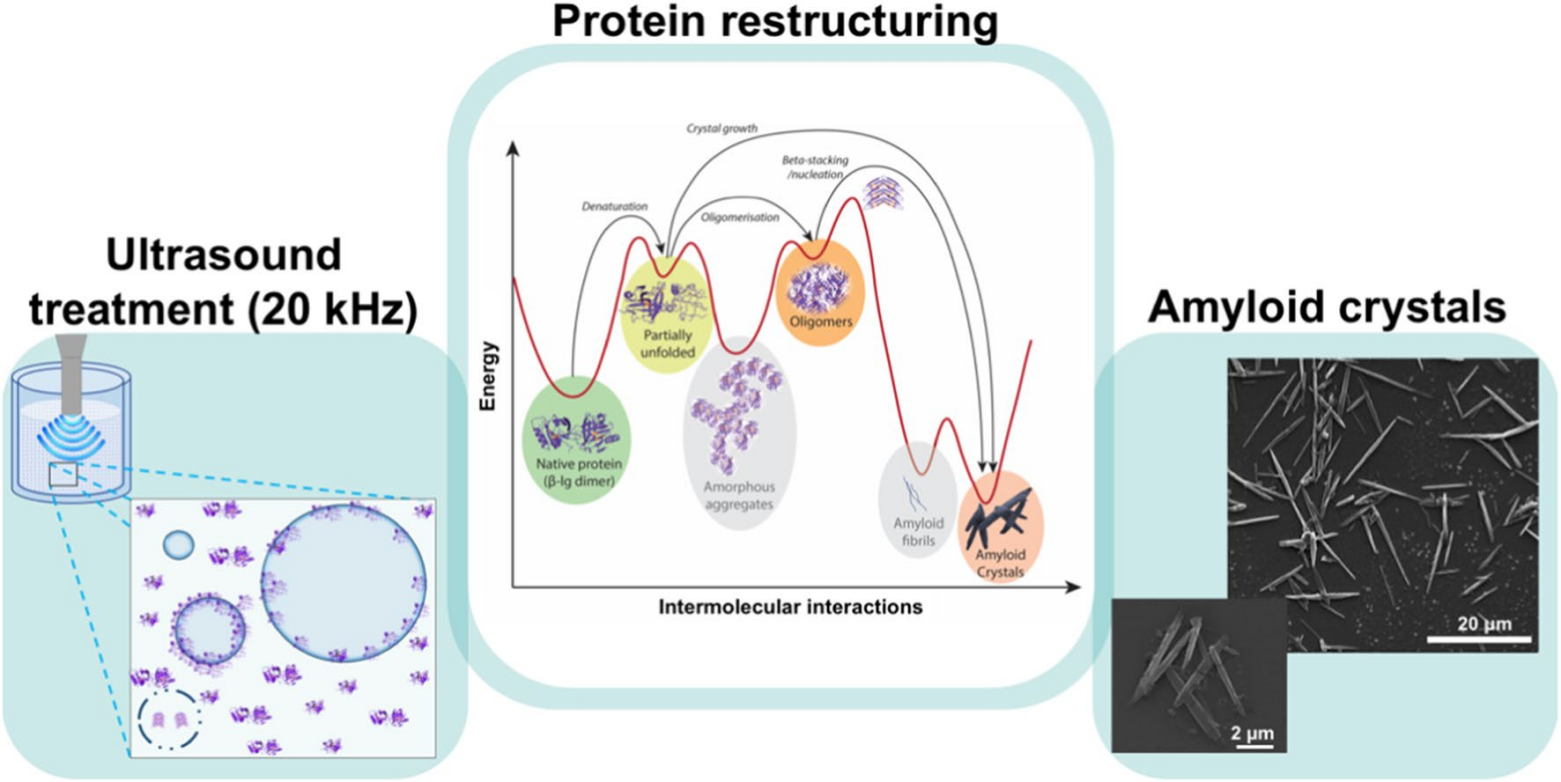

## Introduction

Amyloid structures are β-sheet rich, ordered protein aggregates that are insoluble (Ke et al. 2017). Both biological and synthetically generated amyloids have been actively researched in the past two decades (Knowles and Mezzenga 2016). While efforts are still underway to better understand amyloid formation in neurodegenerative diseases such as Alzheimer’s and Huntington’s disease (Bates and Benn 2002; Glodzik et al. 2012), parallel research has established the potential utility of amyloid structures, outside of their pathological context (Cherny and Gazit 2008). In particular, amyloid structures have gained attention in materials science, with an emphasis in nanobiotechnological applications (Wei et al. 2017), where self-assembly of nanomaterials is a growing research area (Wang et al. 2016). Nanosized functional amyloid structures can serve as scaffolds or can be used in composite materials (Mankar et al. 2011). In bioengineering and cell culture, amyloid scaffolds can be used for cellular growth (Jacob et al. 2015).

To understand the formation of amyloid structures, it is important to closely examine proteins as biomolecules. In vivo, proteins are synthesised in the ribosome, from mRNA (messenger RNA) transcribed from the genes (Nelson and Cox 2008), and later separated from their signal peptide. Following the synthesis of the polypeptide chain, a protein is thermodynamically drawn towards two competing processes of folding and aggregation. Biological systems orchestrate and guide molecular folding to ensure that a newly synthesised protein acquires its functional tertiary form. The intramolecular interactions within a protein molecule are directed towards appropriate folding of the chain into a functional and thermodynamically stable form. However, when the cellular mechanisms that regulate these folding processes are damaged due to aging or genetic anomalies, proteins can fall into aggregation pathways, some of which can result in the formation of amyloid aggregates in vivo (Adamcik and Mezzenga 2018). Outside of their native biological environment, protein molecules can readily interact with each other in ways which allow aggregation. As such, intermolecular protein interactions are typically thermodynamically favoured, with non-native aggregate structures such as amyloids regarded as the most stable thermodynamic energy state (Adamcik and Mezzenga 2018; Balchin et al. 2016). Therefore, while amyloid plaques are associated with disease, amyloid structures are a thermodynamic state of proteins. These ordered amyloid aggregates exhibit structural and molecular polymorphism (Adamcik and Mezzenga 2018). The amyloid fibres/fibrils are flexible and can have varied morphologies such as twisted ribbons or helical ribbons with left/right handedness (Adamcik and Mezzenga 2018). A relatively recent identification of another amyloid polymorph viz. amyloid crystals in in vitro systems has drawn attention for efforts to better understand the energy landscape of amyloid synthesis (Adamcik and Mezzenga 2018; Reynolds et al. 2017). Amyloid crystals are different from conventional purified protein crystals. While aggregation is undesirable for synthesis of conventional protein crystals (McPherson and Gavira 2014), the starting point of amyloid crystals conversely is the ordered aggregation of the partially unfolded proteins/peptides (Adamcik and Mezzenga 2018; Reynolds et al. 2017). This orderliness of amyloid structures (fibrils, fibres, crystals) makes them the thermodynamically favoured state of proteins (Reynolds et al. 2017). However, certain energy barriers must be overcome before native or partially unfolded proteins can achieve that state. A more readily attainable energy state is that of kinetically trapped amorphous aggregates and oligomers that are observed when protein solutions are heated beyond their denaturation temperatures. Therefore, optimised heat or/and shear conditions (Dunstan et al. 2009; Jones et al. 2010; Loveday et al. 2010) are used to drive amyloid synthesis as they help in overcoming the prerequisite energy barriers.

Shear forces can be exerted on a protein in many ways, including by ultrasound-induced cavitation. Ultrasonic processing is an emerging technology for a wide range of applications. Ultrasound are sound waves above the frequency of 20 kHz. The characteristic property of ultrasound processing is acoustic cavitation, which is the formation, growth, and sudden adiabatic implosion of oscillating acoustic bubbles in the medium. Cavitation releases intense energy in the form of heat (> 5000 K), pressure (> 100 atm) and shear in the vicinity of the imploding bubble (Ashokkumar and Mason 2000). The energy is sufficient to produce radical species, which can be used in cross-linking reactions for the formation of protein-based microspheres for encapsulation (Zhou et al. 2010), or for synthesis of functional nanoparticles from cyclic molecules and amino acids (Bhangu et al. 2020). The high-shear microenvironments resulting from high-intensity ultrasound are generated by microjets (100 m/s) and acoustic streaming (10 cm/s) (Ashokkumar and Mason 2000). In an initial study, low-frequency pulsed sonication (ambient temperature, 20 kHz, maximum power 30 W, 5–80 cycles of 5 pulses of 1 s) was shown to form amyloid-like aggregates close to a neutral pH from bovine serum albumin (BSA) and myoglobin (Stathopulos et al. 2004). These aggregates exhibited both amorphous and fibrillar morphologies (Stathopulos et al. 2004).

Ultrasound has been used conventionally to produce uniform seeds for the crystallisation of solutes such as sugars (Dhumal et al. 2008; Zamanipoor and Mancera 2014) or small molecule drugs (Kim and Suslick 2018; Ruecroft et al. 2005). With regard to amyloid synthesis, low-frequency pulsed sonication has been used to induce nucleation for amyloid fibril formation from insulin and α-synuclein proteins (Muta et al. 2014; Yagi et al. 2015), at physiological temperatures of 37 °C and acidic pH. Sonication was used to interrupt the metastability of supersaturated protein solutions to initiate fibril synthesis. Though nucleation was triggered by ultrasound, subsequent fibril formation was not attributed to sonication power, as longer sonication times led to fragmentation of the fibrils (Yagi et al. 2015). Another related study used pulsed ultrasound to break supersaturation to form crystal-like amyloid fibrils from β_2_-microglobulin at low salt concentration, and glass-like amorphous aggregates at higher salt concentrations (Yoshimura et al. 2012). Thus, breaking past supersaturation can induce nucleation for amyloid formation in protein solutions (Noji et al. 2021). In line with the observation that air–water interface is important for amyloid fibrillation at low protein concentrations in vitro (Morinaga et al. 2010), it has been proposed that the cavitation bubble is a nucleation factory (Nakajima et al. 2016), that provides a spherical surface for nucleation for amyloid fibrillation (intermittent sonication of amyloid β peptides at 37 °C, 7.4 pH).

Acoustic cavitation provides catalytic microenvironments (Cavalieri et al. 2016) due to the localised regions of intense heat, pressure and shear generation, that bypass the need for physical catalysts (Lorimer and Mason 1990). Since shear and heat are major drivers of amyloid synthesis (Dunstan et al. 2009; Jones et al. 2010; Loveday et al. 2012), it is possible that high-intensity ultrasound in addition to nucleation could promote amyloid formation and growth. Amyloid formation is a self-assembly phenomenon and its direction is governed by thermodynamics and energy thresholds (Reynolds et al. 2017). In the present work, we hypothesised that the high-shear and high-temperature microenvironments associated with ultrasonic cavitation could be sufficient for both nucleation and for surpassing the energy barriers to drive amyloid formation, even while keeping the bulk fluid close to ambient temperature. Therefore, the motivation of this study was to investigate aggregation and amylogenic potential of β-lactoglobulin (β-lg), a model amyloid protein (Dunstan et al. 2009; Jones et al. 2010; Loveday et al. 2010), using high-intensity ultrasonication in relation to sonication power intensity and solution parameters. The experimental outcome of this work resulted in the formation of β-lg amyloid crystals (Adamcik and Mezzenga 2018; Reynolds et al. 2017). Native whey protein, lysozyme and pea protein isolate (PPI) were also used for certain comparative analyses. Notably, the study explored amyloid synthesis at near native temperatures (ambient or room temperature) and in acidic, neutral and basic pH, to further help understand amyloid crystals and potential pathways for their formation with such solution parameters. The results are encouraging and present an opportunity to further explore potential applications in nanobiotechnology, where fabrication material may require specific synthesis conditions (pH, temperature, etc.) for improved workability.

## Materials and methods

### Materials

Lyophilized bovine β-lg powder (L0130), Thioflavin T (T3516) and lysozyme from chicken egg white (L4919) were purchased from Sigma-Aldrich (Castle Hill, NSW, Australia). Hydrochloric acid (36% w/w), potassium dihydrogen phosphate and dipotassium hydrogen orthophosphate were from Univar (Ingleburn, NSW, Australia). Anhydrous sodium sulfate and sodium hydroxide were from Chem-Supply (Chem-Supply, Gillman, SA, Australia). β-lg was the main protein used in this study as a model protein to investigate its aggregation and amyloidogenic potential with low-frequency ultrasound treatment. It was compared with lysozyme, pea protein isolate and native whey protein to study the effect of using different proteins instead of β-lg. Pisane pea protein isolate (PPI) was purchased from Cosucra (Pecq, Belgium), that was marketed as 88% w/w pea protein. Native whey protein was also used to study the effect of protein concentration on amyloid synthesis, where a higher concentration of β-lg was required (and was a limiting resource), as β-lg makes up 50% of native whey protein (Fox et al. 1998). Native whey protein used was prepared as a part of another study in a 3-stage batch concentration process (Gamlath et al. 2018), by microfiltration of 700 kg of skim milk, followed by permeate collection. Whey protein was concentrated by multistage ultrafiltration (UF) of this permeate, followed by subsequent spray drying (at 80 °C) to obtain a powdered form of native whey protein. Deionized water used for preparation of solutions and buffers in the experiments was taken from Millipore system with 18.2 MΩ cm resistivity.

### Sample preparation

Protein solutions were prepared with some variations for specific experiments. All solutions were filtered with 0.45 µm filters before ultrasound treatment to remove any undissolved particulate substance. The protein and pH of solutions were varied as stated below:

- All β-lg solutions were prepared near their natural concentration value in milk of 3.3 mg/ml (0.33%) (Fox et al. 1998). The typical β-lg sample solutions (pH ~ 7) were prepared in deionised water (Milli-Q). These solutions were used to study the effects of processing time and sonication power on amyloid crystal length. β-lg solutions (3.3 mg/ml) used for ThT fluorescence experiment were prepared in 0.1 M phosphate buffer of pH 7, to avoid assay inconsistencies reported in literature at other pH ranges (Gade Malmos et al. 2017).
- To study the effect of pH variation, the pH of β-lg solutions (3.3 mg/ml) in deionized water was adjusted with 6 M HCl to get a final value of pH ~ 2, and with 1 M NaOH to adjust it to a pH value ~ 10.
- To study the effect of protein type, lysozyme, PPI and native whey protein solutions were prepared at the same concentration as β-lg solutions (3.3 mg/ml) in deionized water. PPI being difficult to completely solubilise, was prepared as a dispersion in deionized water, allowed to stand for 10 min, filtered and used for sonication.
- To study the effect of protein concentration, native whey protein solutions were prepared in deionised water (pH ~ 7) at four different concentrations of 3.3 mg/ml (control), 10 mg/ml, 20 mg/ml and 50 mg/ml, filtered and used.

### Ultrasound treatment

Protein solutions were sonicated in 10 ml volumes for 60 min each, except for the experiments in where effect of processing time was assessed by sonicating samples for 0 min, 5 min, 15 min, 30 min, 45 min and 60 min. The low-frequency ultrasound (20 kHz) was used to treat the samples with an 11 mm tip transducer at 20 kHz (Branson Ultrasonics, USA, Model No. 450, 400 W). The standard low-frequency treatment was performed at 50% amplitude that translated to an input power of 200 W. To understand the effect of ultrasonic power on synthesis, the amplitude was varied (10–60%) to provide input power density between 4 and 24 W/cm^3^, keeping sample preparation time constant of 60 min each. All the sonication treatments were performed in a jacketed vessel and the temperature was maintained with a water bath running at 20 ± 1 °C. Samples were prepared in duplicate.

### Non-reducing sodium dodecyl sulfate polyacrylamide gel electrophoresis (NR-SDS-PAGE)

Bio-Rad pre-cast 4–20% gradient criterion gels (Bio-Rad, Gladesville, NSW, Australia) were used for analysis by sodium dodecyl sulfate polyacrylamide gel electrophoresis (SDS-PAGE). To have a protein concentration suitable for to PAGE analysis, the sonicated protein samples (β-lg, 3.3 mg/ml in deionised water, pH ~ 7) were diluted to 1 mg/ml. Each of the samples was then mixed with an equal volume of Lamelli Sample Buffer (Bio-Rad, Gladesville, NSW, Australia). Running buffer was prepared by 1:10 dilution of Tris–glycine SDS Buffer (Bio-Rad, Gladesville, NSW, Australia) for SDS-PAGE with deionized water. The single well volume of the pre-cast gel was 30 μl. Hence, 20 μl of each sample was loaded into separate wells. Electrophoresis was performed at a constant voltage of 200 V for 42 min in a Criterion™ cell (Bio-Rad, Gladesville, NSW, Australia). After the run, the gels were washed three times for 5 min each with deionized water using gentle shaking. They were then left in 60 ml of Bio-safe Coomassie stain on a table-top shaker overnight (~ 12 h) for staining. Stained gels were then rinsed twice and placed in 100 ml of deionized water for 6 h with gentle shaking for removal of excess stain. The gel was imaged using a Bio-Rad Gel Doc XR and Imager. ImageJ software was used to for gel densitometry analysis to quantify protein band intensities for relative comparison (Carter et al. 2013).

### Optical microscopy

The sonicated samples were mounted between glass slide and cover slip and observed in bright field mode with a 60 × oil emersion objective lens under an inverted Olympus IX71 (Olympus, Macquarie Park, NSW, Australia) wide field fluorescence optical microscope, using oil of 1.54 RI.

### Scanning electron microscopy

Sonicated samples were prepared for imaging on 1 × 1 cm diced silicon wafers, with two rounds of washing with deionized water, followed by drying inside a closed box. Samples were then sputter coated with a thin layer of gold and observed by high-resolution field emission environmental scanning electron microscope in high vacuum at 10.0 kV with Everhart–Thornley (ED) detector (Quanta 200 FEI, Thermo Fisher Scientific, Australia).

### Scanning helium ion microscopy

Sonicated samples were prepared for imaging on silicon wafers, with two rounds of washing with deionized water, followed by drying inside a closed box. The samples were then imaged using helium ion microscopy (HIM) on an ORION NanoFab (Zeiss, Peabody, USA). A 25 keV He^+^ beam was used as the probe with a typical current of 1 pA. The secondary electron signal was acquired by an Everhart–Thornley (ET) detector, and images were collected in line averaging mode with 64 averages and a pixel dwell time of 1 μs. Images were collected at a stage angle of 30°.

### Measurement of fluorescence with Thioflavin T

Stock solution of Thioflavin T (ThT) of 0.05 M concentration was prepared in deionized water and stored in dark under refrigeration and was suitable to be used within a week. Thioflavin T stock solution was added to sonicated protein solutions (β-lg, 3.3 mg/ml in 0.1 M phosphate buffer, pH ~ 7) to give a final dilution of 250 µM (Dunstan et al. 2009), which were used for measurement of fluorescence at 482 nm (λ_ex_ = 440 nm, λ_em_ = 482 nm)$.$ Fluorescence was measured using Shimadzu RF-5301PC fluorescence spectrophotometer fitted with a xenon lamp. A slit width of 5 nm was used for both excitation and emission spectra, and the optical length of the quartz cuvette was 1 cm. All the protein samples for this assay were prepared in phosphate buffer (pH 7) to avoid inconsistencies reported in literature at other pH ranges (Gade Malmos et al. 2017).

### Circular dichroism (CD) spectroscopy

Far-UV circular dichroism (CD) spectra of protein solutions were recorded from 190 to 260 nm on an AVIV CD-spectrometer (Biomedical Inc., New Jersey, USA). Solutions were diluted before measurement to give a final concentration of 0.3 mg/ml. The path length was 1 mm, the spectral resolution was 1 nm, data collection interval and bandwidth were 1 s and 1 nm, respectively (Devnani et al. 2020). Samples were prepared in duplicate and each recorded spectrum was an average of 3 scans.

### Size exclusion chromatography

Size exclusion chromatography (SEC) was performed using Shimadzu SCL–10AVP high performance liquid chromatography (HPLC) equipment, fitted with Tosoh Bioscience size exclusion column TSKgel G3000SWXL for protein separation, and the UV detector set at 280 nm. SEC protocol was performed at isocratic elution with a solution of sodium sulfate and potassium phosphate salts (Na_2_SO_4_ 0.1 M, K_2_HPO_4_ 0.1 M, NaN_3_ 0.05%) at a flow rate of 1 ml/min. The sample injection volume was set to 20 µl. The chromatograms were obtained using LabSolutions software from Shimadzu.

### Polarised optical microscopy

Olympus cross polarisers mounted to the existing optical microscopy set up were used and the imaging was done at variable angles to record crystal birefringence.

### Fourier transform infrared (FTIR) spectroscopy

A PerkinElmer Spectrum 100 FTIR Spectrometer (PerkinElmer, Melbourne, Australia) was used to record the FTIR spectra of protein solutions (β-lg, 3.3 mg/ml in deionised water, pH ~ 7). The spectra were obtained in the range from 4000 to 650 cm^−1^, with subtracted background. Post spectral measurement, the data was processed using Origin software (OriginLab Corporation, Northampton, USA). Each transmittance signal was processed as a 2° derivative between the range 1700–1600 cm^−1^ and processed further by curve fitting for identification of peaks relevant to the secondary structure of proteins in broad amide I region (Yang et al. 2015). Samples were prepared in duplicate and each spectrum was recorded at a resolution of 4 cm^−1^ and an average of 128 scans to improve the signal to noise ratio.

### ImageJ analysis of length of amyloid protein structures

The SEM images of the amyloid structures were studied with ImageJ software developed at the National Institutes of Health (NIH), USA. The Fiji add-in was used, and the amyloid crystals were measured to scale with the help of the measure tool. The software also provided the statistical capability to process the data. The samples were prepared in duplicate and the image data were collected over triplicate experiments. ImageJ was also used for gel densitometry analysis for NR-SDS-PAGE (Carter et al. 2013; Pathak et al. 2020).

## Results and discussion

**Fig. 1:**
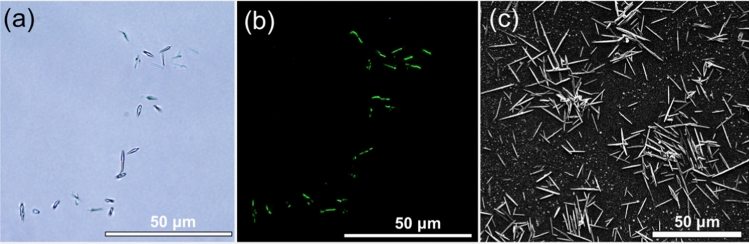
Well-defined mesoscopic amyloid crystals formed after sonication of pure β-lactoglobulin solutions. **a** Clusters of defined mesoscopic aggregate structures observed under bright field microscopy; **b** same cluster panel in fluorescent green under blue–violet excitation fluorescence filter; and **c** SEM image of aggregates showing clear acicular crystals. (All the samples imaged here were sonicated with 20 kHz ultrasound for 45 min at 20 W/cm^3^ input power density, 20 ± 1 °C, pH 7.0 ± 0.4. Scale bars are 50 μm)

Low-frequency ultrasound produces high shear and high temperature microenvironments (Ashokkumar and Mason 2000) capable of inducing protein aggregation (Chandrapala et al. 2011). Initial experiments were performed to investigate if 20 kHz ultrasound could produce either disordered aggregates or ordered aggregates from β-lg. Although no visible change was observed in the β-lg solutions, with the solutions remaining clear even after prolonged sonication, microscopic imaging revealed the formation of aggregates. Microscopic characterisation showed protein aggregates with a defined short fibril-like structural outline in the mesoscopic size range (Fig. 1). The characteristic amyloid autofluorescence (Apter et al. 2020) was evident for these amyloid crystals from observations made with fluorescence optical microscopy (Fig. 1b). SEM images confirmed these aggregates to be acicular (needle-like) amyloid crystals (Fig. 1c).

### Effect of sonication parameters on amyloid formation from β-lactoglobulin

**Fig. 2:**
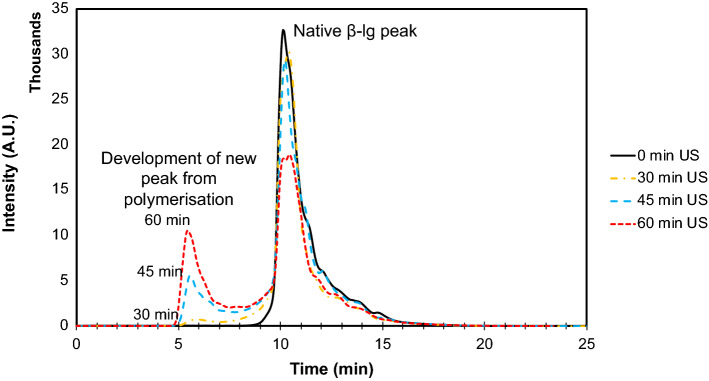
Size exclusion chromatography for measuring conversion of native β-lg protein. SEC chromatograms show the formation of higher molecular weight moieties and depletion of native protein with increasing sonication time. All the samples were sonicated with 20 kHz ultrasound at 20 W/cm^3^, 20 ± 1 °C, pH 7.0 ± 0.4

Processing time and power density are important sonication factors and hence a series of experiments was performed to investigate amyloid formation in relation to these variables. Size exclusion chromatography was performed to assess the relative amount of soluble native protein and amyloid protein present in the samples as a function of sonication time. The chromatograms show a decrease in the native protein peak (11 min) and the formation and growth of a new peak at increased molecular weight (6 min), reflective of the amyloid crystals, as a function of sonication time (Fig. 2).

**Fig. 3:**
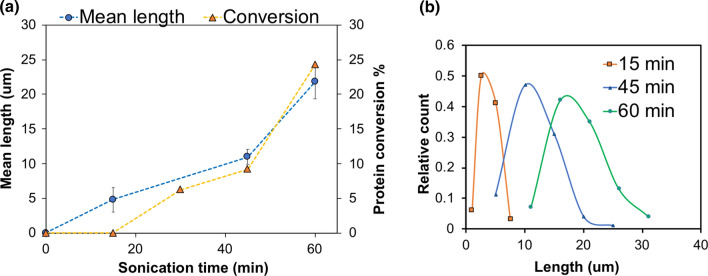
Protein conversion corresponds to increase in mean crystal length and polydispersity. **a** Length measurements obtained from SEM images were compared against the conversion percentage of native protein into higher molecular weight moieties. Conversion was calculated based on the depletion of the native β-lg peak. **b** Amyloid crystal length distribution for sonicated samples show an increase in polydispersity with increasing sonication time. All the samples were sonicated with 20 kHz ultrasound at 20 W/cm^3^ input power density, 20 ± 1 °C, pH 7.0 ± 0.4. Data and error bars are the average and standard deviation of mean length values determined from measurements of 100 crystals for each treatment, from triplicate experiments

It was observed that about 25% of the native protein was converted in 60 min of ultrasound processing. The conversion percentage of native β-lg as a function of sonication time was compared to the increase in mean length of amyloid crystals formed (Fig. 3a). SEM images were analysed to determine the mean length of amyloid crystals, which were already starting to form in samples sonicated for only 15 min. For samples of β-lg sonicated at 20 W/cm^3^ input power density, the mean crystal length increased with sonication time from 4.7 μm at 15 min to 10.8 μm at 45 min, and 21.8 μm after 60 min (Fig. 3a). Although an immeasurably small amount of protein had been converted after 15 min of sonication (SEC, Fig. 2), some small crystals (~ 5 µm) had already been formed. The subsequent increase in protein conversion was proportionally greater than the increase in length, suggesting that new amyloid crystals continued to be formed as the existing crystals grew. The same observation can be confirmed from the amyloid crystal length distribution (Fig. 3b), which shows an increase in polydispersity over time. This suggests that nucleation continued to occur during the process, and that crystal extension was slow and progressive. Protein denaturation due to ultrasound leads to an increase in surface hydrophobicity and protein–protein interactions (Chandrapala et al. 2011), which can form nuclei for amyloid crystals. Acoustic cavitation from ultrasound also creates conditions for saturation of denatured/unfolded proteins at the bubble interface (Cavalieri et al. 2008) to enable the rapid formation of amyloid crystal nuclei in the solution. The reason for the apparent increase in rate of crystal extension at 45–60 min is not clear but it could be due to the combined effect of a decrease in the rate of new crystal formation and the continued growth of existing crystals, which work together to increase the mean length (Figs. 2, 3).

**Fig. 4:**
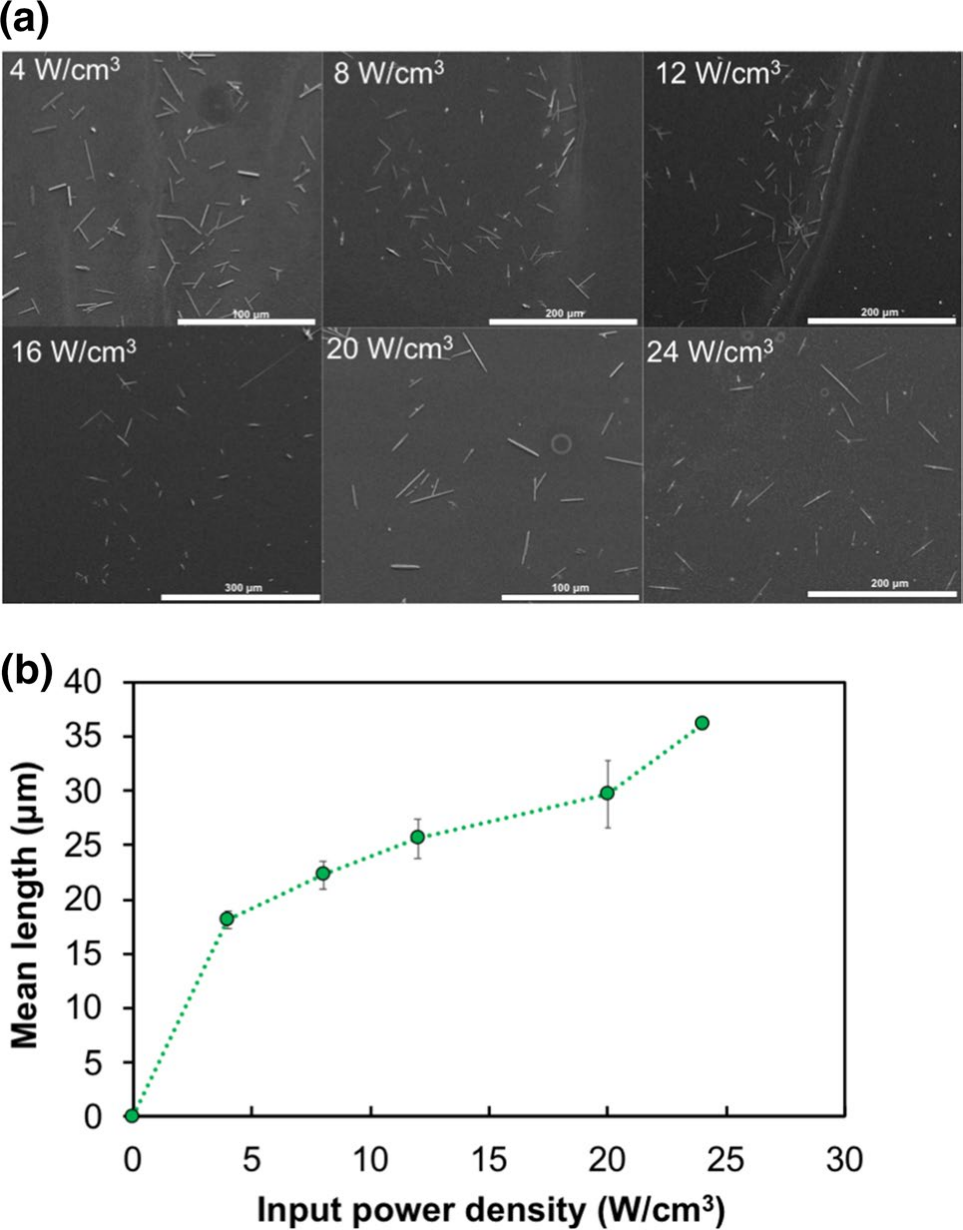
Effect of sonication power on amyloid formation. **a** Representative SEM images of crystals formed at increasing power densities of ultrasound treatment for constant processing time of 60 min. Scale bars are 100, 200, 200, 300, 100 and 200 μm, respectively. **b** Increase in the mean length of amyloid crystals with an increase in input power density for 10 ml sample volume. All the samples were sonicated with 20 kHz ultrasound at 20 ± 1 °C and pH 7.0 ± 0.4. Data and error bars are the average and standard deviation of mean length values determined from measurements of 100 crystals for each treatment, from triplicate experiments. (16 W/cm^3^ is not reported in 4(**b**) due to insufficient data points from triplicate experiments (i.e., < 100 crystals) to represent mean length. The mean lengths for 20 W/cm^3^ treatment differ in Figs. 3a and 4b due to experimental variations)

The effect of ultrasound treatment is dependent on the physical characteristics of the equipment and operation. The equipment used for sonication was an 11 mm horn, which can be considered to deliver the ultrasound power at a focussed region in the solution. The input power density of the applied ultrasound is dependent on the power amplitude of the system and the volume of sample liquid being processed. For β-lg solutions treated with low-frequency (20 kHz) for constant processing time of 60 min, the mean length of the amyloid crystals increased with an increase in input power density from 4 W/cm^3^ to 24 W/cm^3^ (Fig. 4). The minimum power density of 4 W/cm^3^ for the current experimental setup was found sufficient to convert β-lg protein into crystal-like ordered aggregates. It could be observed (Fig. 4b) that the mean length of crystals formed was not linearly proportional to the input power, and at power levels above 20 W/cm^3^, the slope was comparatively steeper than in the mid-power range, indicating a proportionally greater increase in length could be achieved at high power levels.

### Effect of solution pH on ultrasound-induced amyloid crystal formation

**Fig. 5:**
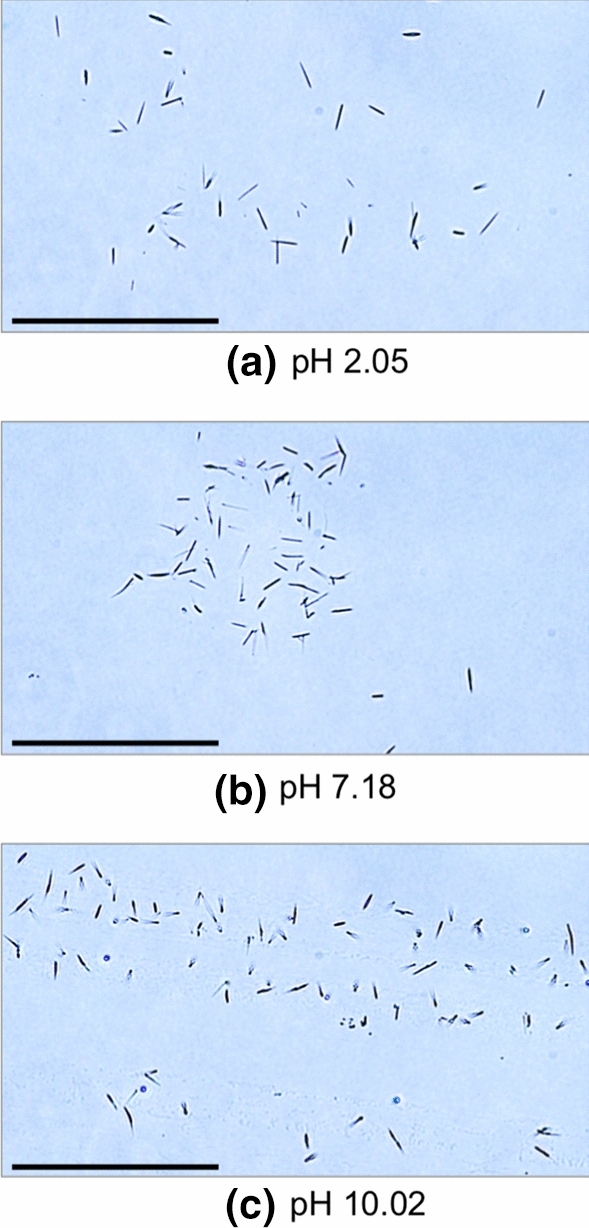
β-lg amyloid crystals of synthesised by sonication at different pH. Bright field optical microscopy images show amyloid crystals formed at 20 W/cm^3^ for 60 min treatment in solutions at different starting pH **a** 2, **b** 7, and **c** 10. All the samples were sonicated at 20 kHz, 20 ± 1 °C and pH 7.0 ± 0.4. Scale bars are 50 μm

Most in vitro model systems studied for the synthesis of β-lg fibrils were acidic between pH 1 and 3 (Dunstan et al. 2009; Jones et al. 2010; Loveday et al. 2010). The isoelectric point (pI) of bovine β-lg exists at pH 5.1. The protein is monomeric at pH 2, forms an octamer between pH 3.5–5.2 (peaking near pH 4.5), exists as a dimer near physiological pH 7, and acquires a tetrameric form above pH 7.5 (Kelly et al. 2009). Since the monomer–dimer transition of β-lg favours the monomeric form below pH 3.5 (Sawyer 2003), acidic model systems were used to allow the protein to initiate the fibril synthesis as an unfolded monomer. Though some studies have investigated synthesis of β-lg fibres at near neutral pH, such solutions contained urea or guanidinium chloride to enable protein unfolding (Hamada and Dobson 2002), or polysaccharides (Ma et al. 2013) to cause macromolecular crowding. Some other studies that have reported the effect of pH on the formation of β-lg amyloid fibrils encountered spherical particles near the pI (pH 5.1) and neutral pH values (Nicolai et al. 2011). In the present study, amyloid formation was attempted at pH ~ 7 (neutral, β-lg primarily dimeric), pH ~ 2 (acidic, β-lg primarily monomeric) and pH ~ 10 (basic, β-lg primarily tetrameric). The results demonstrate that amyloid crystals can be produced by ultrasonication regardless of the solution pH (Fig. 5). The ability to produce amyloid crystals at different pH values with similar morphology indicates that initial the tertiary/quaternary protein structure did not affect the aggregate formation. This could be attributed to the partial unfolding of proteins due to extreme local shear created by ultrasound irrespective of solution pH (Chandrapala et al. 2012; Villamiel and de Jong 2000).

### Effect of protein type and concentration on ultrasound-induced amyloid crystal formation

**Fig. 6:**
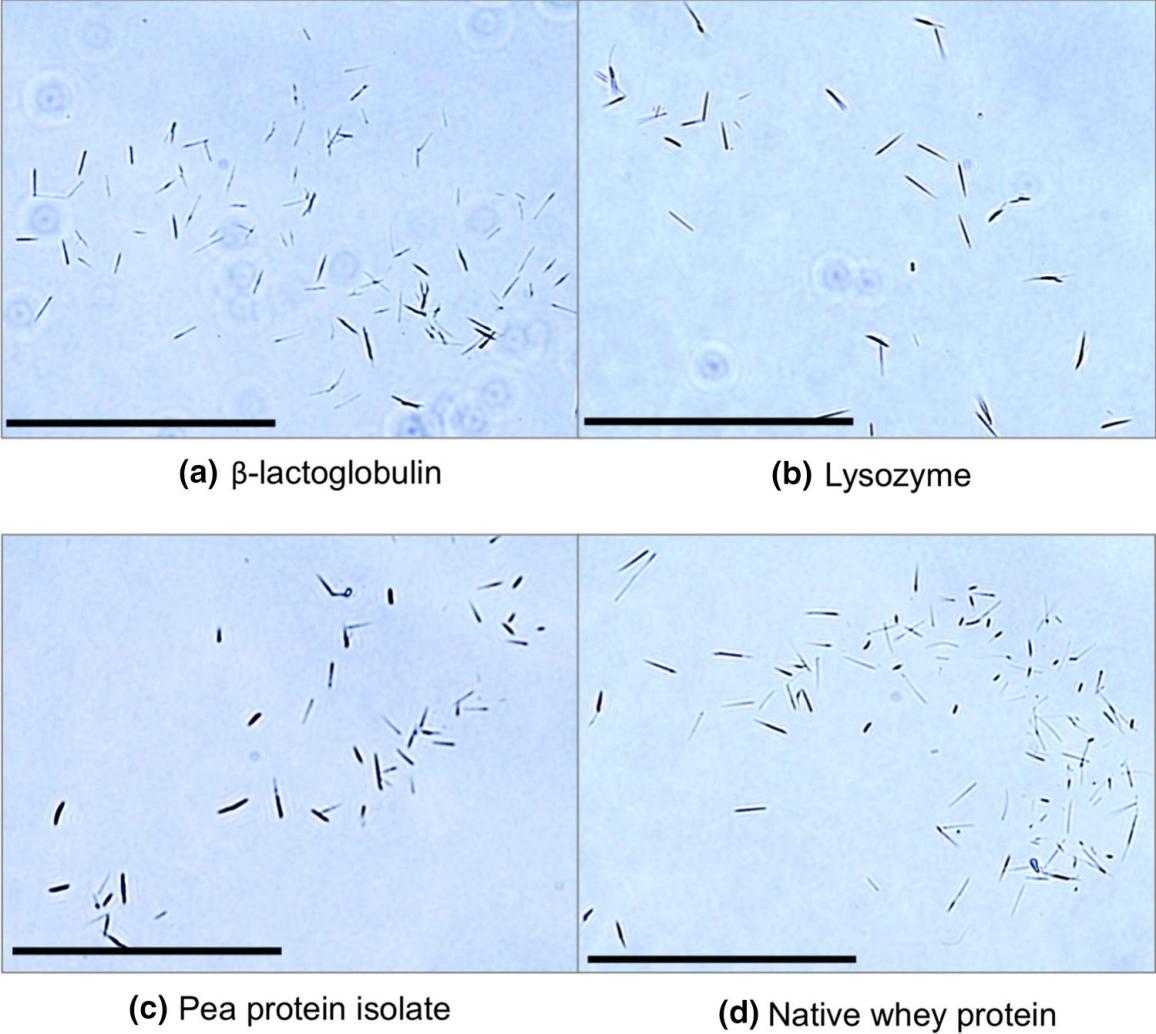
Amyloid crystals prepared with different proteins. **a** β-Lactoglobulin, **b** lysozyme, **c** PPI, and **d** native whey protein. All the samples were sonicated with 20 kHz ultrasound at 20 W/cm^3^ input power density for 60 min, 20 ± 1 °C, pH 7.0 ± 0.4. Scale bars are 50 μm

To investigate if the observed behaviour was characteristic of β-lg or more universal (Guijarro et al. 1998; Noji et al. 2021), experiments were performed on individual proteins (β-lg and lysozyme) and protein mixtures (pea protein isolate (PPI) and native whey protein). For the proteins and mixtures used in this study, amyloid synthesis was achieved by low-frequency ultrasound (20 kHz), resulting in amyloid crystals with similar morphologies (Fig. 6).

Lysozyme and β-lg have eight and five cysteine residues, respectively, and can form intermolecular disulfide bonds when activated by ultrasound (Cavalieri et al. 2008). Native whey protein and PPI also contain cysteine residues. The cysteine residues in proteins form disulfide bonds, including intermolecular cross-links, if a sulfhydryl group (-SH) is available for reaction. Such cross-links may be involved in amyloid formation (Li et al. 2013); however, they are not a prerequisite (Mossuto et al. 2011). A growing view on amyloids is that it is a common structural property of proteins (Hoppenreijs et al. 2021), specially globular proteins (Guijarro et al. 1998), and under optimum conditions most proteins can form amyloid structures. The non-covalent interactions, predominantly hydrophobic interactions and hydrogen bonding, are credited for β-sheet stacking forming an ordered structure (Schleeger et al. 2013). The generic interbackbone hydrogen bonding network is mainly responsible for the rigidity/strength of amyloid fibrils (Knowles et al. 2007). Thus, the common β-stacking feature observed in all amyloid aggregates is not because of shared sequences among proteins, instead it is due to the common non-covalent interactions (Schleeger et al. 2013). These insights into amyloid chemistry thus explain the similar morphologies obtained from ultrasound-induced formation of amyloid crystals using different proteins.

**Fig. 7:**
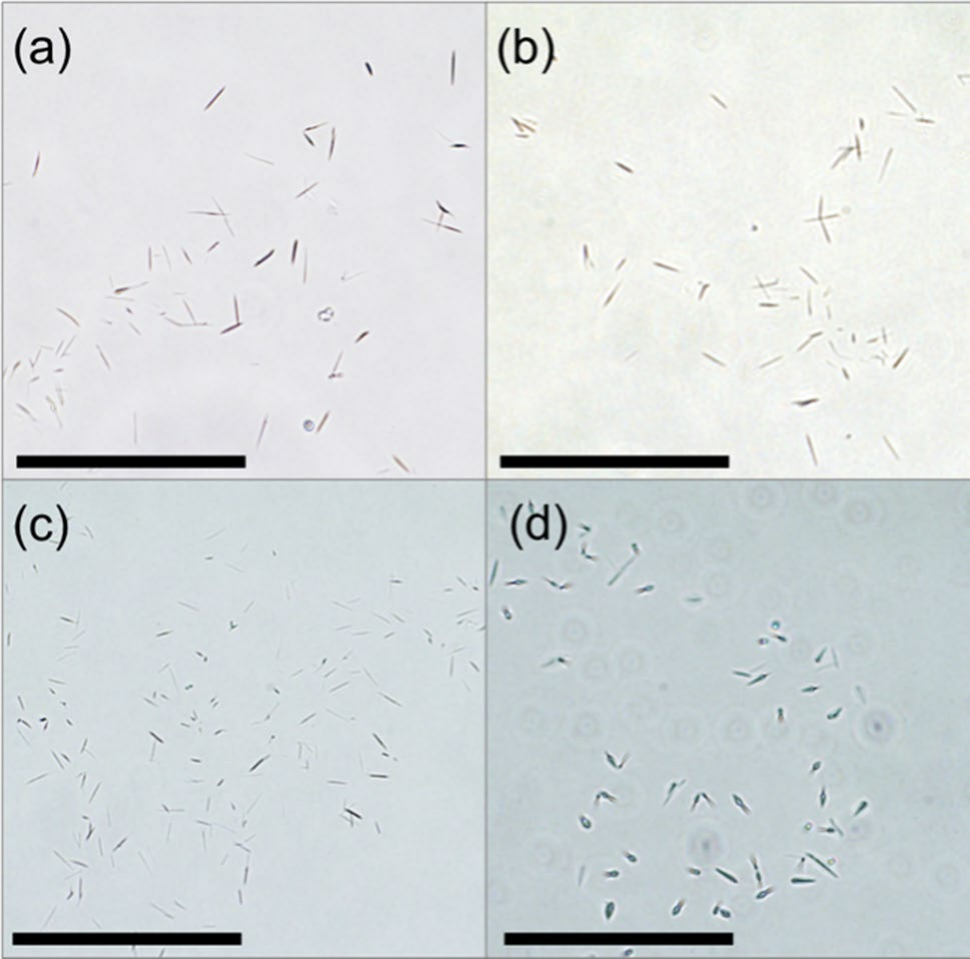
Concentration tolerance for amyloid crystal synthesis using ultrasound. Bright field optical microscopy images of native whey protein solutions show similar crystal growth with 20 kHz ultrasound treatment at 20 W/cm^3^ input power density for 60 min, 20 ± 1 °C, 7.0 ± 0.4 pH, at different native whey protein concentrations of **a** 3.3 mg/ml, **b** 10 mg/ml, **c** 20 mg/ml, and **d** 50 mg/ml. Scale bars represent 50 μm

Proteins are known to aggregate at higher concentrations on account of decreased inter-protein distance and increased “crowding” (Wang and Roberts 2018). The formation of amorphous aggregates and ordered aggregates can be considered competing pathways, the relative tendency of which can be affected by protein concentration. An increased protein concentration may favour the formation of amorphous aggregates over amyloid crystals (Chiti et al. 2000, 1999; Yoshimura et al. 2012), and their formation is kinetically controlled (Yoshimura et al. 2012). To investigate this, native whey protein solutions (where in β-lg makes up 50% of the protein content (Fox et al. 1998)) of different concentrations (3.3–50 mg/ml) were sonicated for 60 min at 20 W/cm^3^. Amyloid crystals were observed in all concentration variations, but amorphous aggregates were not formed (Fig. 7). This indicates that ultrasonically driven formation of amyloid crystals is favoured over amorphous aggregation over a practically significant tolerance range of protein concentrations.

### Amyloid crystal growth and structure

**Fig. 8:**
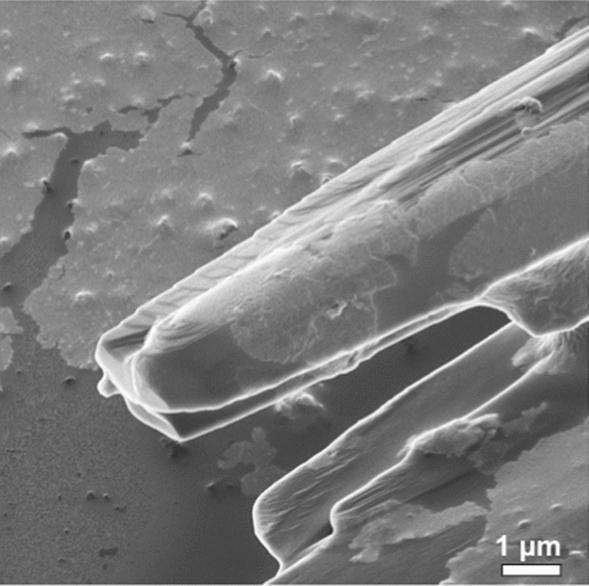
Physical structural details of β-lg amyloid crystals. The wafer-like layered arrangement in amyloid crystal formed by sonication as seen under helium ion microscopy, scale bar is 1 μm. The sample was sonicated at 20 kHz for 60 min at 20 W/cm^3^ input power density, 20 ± 1 °C, pH 7.0 ± 0.4

As demonstrated, amyloid crystals can be produced from ultrasonication of β-lg and other proteins over a range of concentrations, pH values and ultrasonic power intensities. Structural characterisations were performed on the transition of β-lg into amyloid crystals during ultrasonication to understand the mechanism of formation of β-lg amyloid crystals and the preference for crystal structures over fibrils and amorphous aggregates. Detailed SEM images of the amyloid crystals were obtained to probe their microstructure. The crystals have a needle-like structure that tapers to an irregular end with a wafer-like, layered structure (Fig. 8). This structure indicates that the physical growth of the crystals occurs via a layer-by-layer deposition, with preferential addition of protein material along the longitudinal axis as compared to the cross section. This would result in linear growth leading to an increase in the aspect ratio (length: width).

**Fig. 9:**
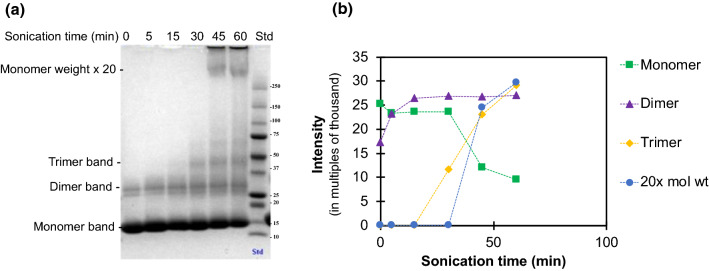
Non-reducing SDS-PAGE of sonicated β-lg solutions. **a** Samples in increasing order of sonication time from left to right (0–60 min). A steady increase is seen in higher molecular weight aggregates, with high-intensity bands at multiples of monomeric β-lg (MW = 18.4 kDa) including dimer and trimer bands. **b** Quantitative estimation of relative changes in the bands of same molecular weight from this gel. All samples were treated with 20 kHz ultrasound at 20 W/cm^3^ input power density, pH = 7.0 ± 0.4 and T = 20 ± 1 °C

To investigate the possible size of the repeating units depositing onto the crystals, non-reducing SDS-PAGE (NR-SDS-PAGE) analysis of β-lg solutions (in which the disulfide bonds remain intact) was performed as a function of processing time low-frequency ultrasound treatment (20 kHz, 20 W/cm^3^, pH = 7.0 ± 0.4, T = 20 ± 1 °C). The results (Fig. 9) revealed an increase of higher molecular weight bands in NR-PAGE accompanied by a proportionate decrease in the intensity of the 18.4 kDa monomeric band. During the first 30 min of sonication, there was an increase in the intensity of the dimeric and the emergence and progressive increase in trimeric bands. Subsequently, higher molecular weight bands, including an extra band corresponding to a molecular weight ~ 20 times that of monomeric β-lg, were noticeable after 45 min of sonication. These results indicate a progressive series of aggregation events, with initial polymerisation or seeding, preceding the formation of larger aggregates of much higher molecular weights, which can be presumed to be the amyloid crystals or precursors. It is interesting to note that although high molecular weight aggregates in PAGE were only observable after 45 min (Fig. 9), small amyloid crystals were already present in samples ultrasonicated for 15 min (Fig. 3). Consistent with the above results (Fig. 3), this suggests that individual crystals can form quickly relative to the rate of nucleation and overall protein conversion. This indicates that changes in the protein structure that lead to nucleation may be the rate-limiting step.

### Alterations in protein secondary structure during amyloid crystal formation

**Fig. 10:**
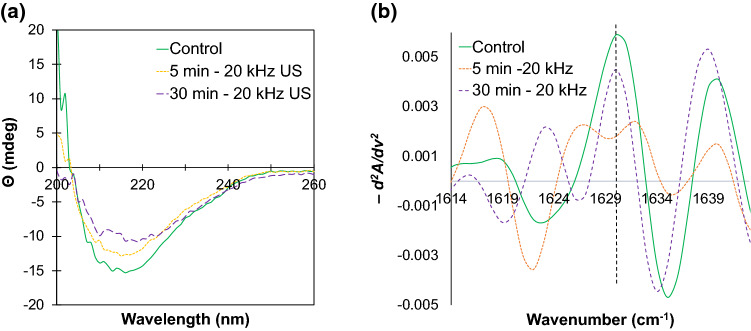
CD and FTIR measurements of β-lactoglobulin amyloid crystals. **a** CD spectra for β-lactoglobulin solutions show an overall decrease in molar ellipticity signal indicating an overall decrease of protein in solution due to aggregation. **b** Secondary derivative of the FTIR spectra show the development of two peaks from an initial single peak at 1630 cm^−1^_,_ indicating an increase in antiparallel β-sheet content due to intermolecular stacking. All the samples were sonicated at 20 kHz at 20 W/cm^3^, 20 ± 1 °C, pH 7.0 ± 0.4

To further develop an understanding of the mechanism of amyloid crystal formation with low-frequency ultrasound, structural analysis of β-lg was performed using far-UV circular dichroism (CD) spectroscopy. The structural peaks in far-UV CD spectra of β-lg solutions remained relatively constant following ultrasound treatment (Fig. 10a). The secondary structure of β-lg is rich in β-sheet (38–46%) (Chandrapala et al. 2012; Kim et al. 2005), and the peak at around 216–218 nm, which corresponds to the *n* to π* transitions for β-sheet, confirms that β-sheet remained the major structural element (Kelly et al. 2005) in all samples. However, there was an observable decrease in the molar ellipticity upon sonication (Fig. 10a). This could be attributed to the aggregated β-lg moving out of solution, since CD spectroscopy only measures the proteins in solution, with aggregated proteins falling out of detection (Ioannou et al. 2015).

FTIR analysis was performed to provide more detailed information about the changes in the β-sheet structure of β-lg due to sonication. The secondary derivatives of FTIR spectra of β-lg solutions (Fig. 10b) show that ultrasound treatment caused the β-sheet peak at 1630 cm^−1^ to split and create two resultant peaks at around 1627 cm^−1^ that correspond to antiparallel β-sheet structures. This suggests that the protein molecules formed stronger intermolecular β-sheet structures, moving from a native β-sheet state to a more compact or dense antiparallel β-sheet configuration (Ioannou et al. 2015). This change could be associated with either the progressive polymerisation of the protein (Fig. 9) or the amyloid crystals themselves, which had begun to form within this time period (Fig. 3) and which are able to contribute to the FTIR signal.

**Fig. 11:**
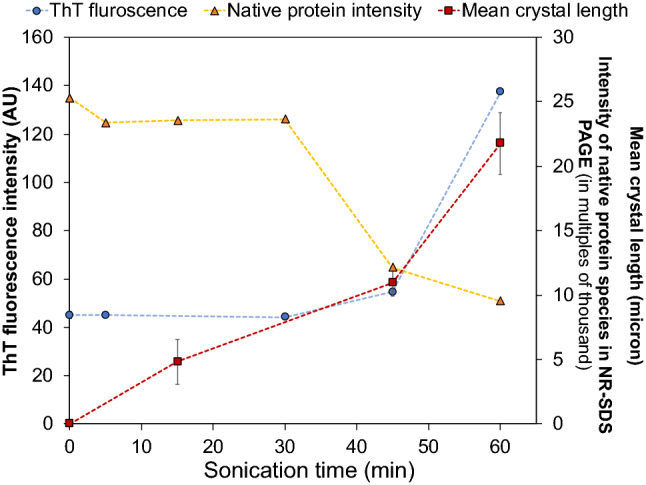
ThT fluorescence assay. Measurement of fluorescence with ThT (λ_ex_ = 440 nm, λ_em_ = 482 nm) compared to the increase in mean crystal length and decline of native protein band intensity from NR-SDS-PAGE. Emission intensities for fluorescence with ThT show a lag phase followed by increased beta-stacking. This is in correspondence to the observed increase in the mean length of amyloid crystals, and the decline in native protein intensity (NR-SDS-PAGE). These trends demonstrate a clear transition from the lag phase into the growth phase. All the samples were sonicated at 20 kHz at 20 W/cm^3^, 20 ± 1 °C, pH 7.0 ± 0.4

Thioflavin T (ThT) fluoresces upon binding to a β-sheet region of protein molecules and is used to investigate an increase in β-stacking during amyloid formation (Dunstan et al. 2009). As the amyloid formation increases, there is an increase in fluorescence intensity (Dunstan et al. 2009). As shown in Fig. 11, the fluorescence assay with ThT showed an initial lag followed by an increase in fluorescence intensity during sonication, confirming an increase in β-stacking as a result of ultrasound processing. When compared with the intensity of native β-lg, ThT fluorescence showed an inverse relationship confirming that depletion in native protein was accompanied by an increase in β-stacking. The apparent lag in ThT fluorescence (30–45 min) beyond the time when crystals were first detectable microscopically (15 min; Fig. 3) reflects the low number of crystals that were formed initially, from which insufficient signal could be generated to be detected by this method. After 45 min, the rapid increase in ThT fluorescence was similar to the trend observed for the increase of mean fibril length.

β-lg is a globular protein that consists of nine β-sheet strands (of which eight are antiparallel) (Brownlow et al. 1997) which have the potential to undergo structural transformations to allow for beta stacking and formation of amyloid fibrils. CD and FTIR spectroscopic analysis of the changes in the secondary structure of β-lg following low-frequency ultrasound treatment (Fig. 10) showed that sonication produced protein aggregates richer in antiparallel β-sheet structures, which is one of the characteristic features of amyloid fibrils (Schladitz et al. 1999). The NR-SDS-PAGE (Fig. 9) and ThT fluorescence assay (Fig. 11) results indicated small change in the protein until 45 min of sonication. In contrast, amyloid crystals could be observed microscopically after only 15 min of sonication (Fig. 3). From these results it can be deduced that crystal growth can proceed rapidly compared to the nucleation and protein conformation changes, which can be inferred as rate-limiting, as opposed to mass transfer and deposition of denatured proteins onto existing crystals. These complementary observations support the early inference that the β-lg amyloid crystal formation observed in this study follow a nucleation mechanism, which is another characteristic of amyloid structures (Adamcik and Mezzenga 2018).

To summarise, the β-lg amyloid crystals formed in this study exhibited crystal morphology (SEM), along with autofluorescence (OM); crystallization kinetics of growth (SEC, NR-SDS-PAGE); increased antiparallel β-sheet content (CD, FTIR); dramatic increase in β-stacking (ThT fluorescence); and presence of polymeric protein aggregates with increased molecular weight (NR-SDS-PAGE), all characteristics that are consistent with known properties of amyloid structures (Apter et al. 2020; Dunstan et al. 2009; Martins et al. 2020; Reynolds et al. 2017). X-ray diffraction data, however, is required to characterize the crystallinity and the amyloid nature of the structures.

### Proposed mechanism for the formation of amyloid crystals

The synthesis of amyloid crystals could be explained based on the known properties of sonochemical systems, wherein intermolecular interactions are favoured at the acoustic bubble-solution interface and increased mass transfer is observed due to acoustic cavitation (Ashokkumar and Mason 2000). As amyloid synthesis follows the mechanism of crystallisation (Sheftic et al. 2009; Yoshimura et al. 2012), drawing on the well-established concept of sonocrystallisation (Kim and Suslick 2018), could help to elaborate on the mechanism of ultrasound-induced amyloid crystal formation.

**Fig. 12:**
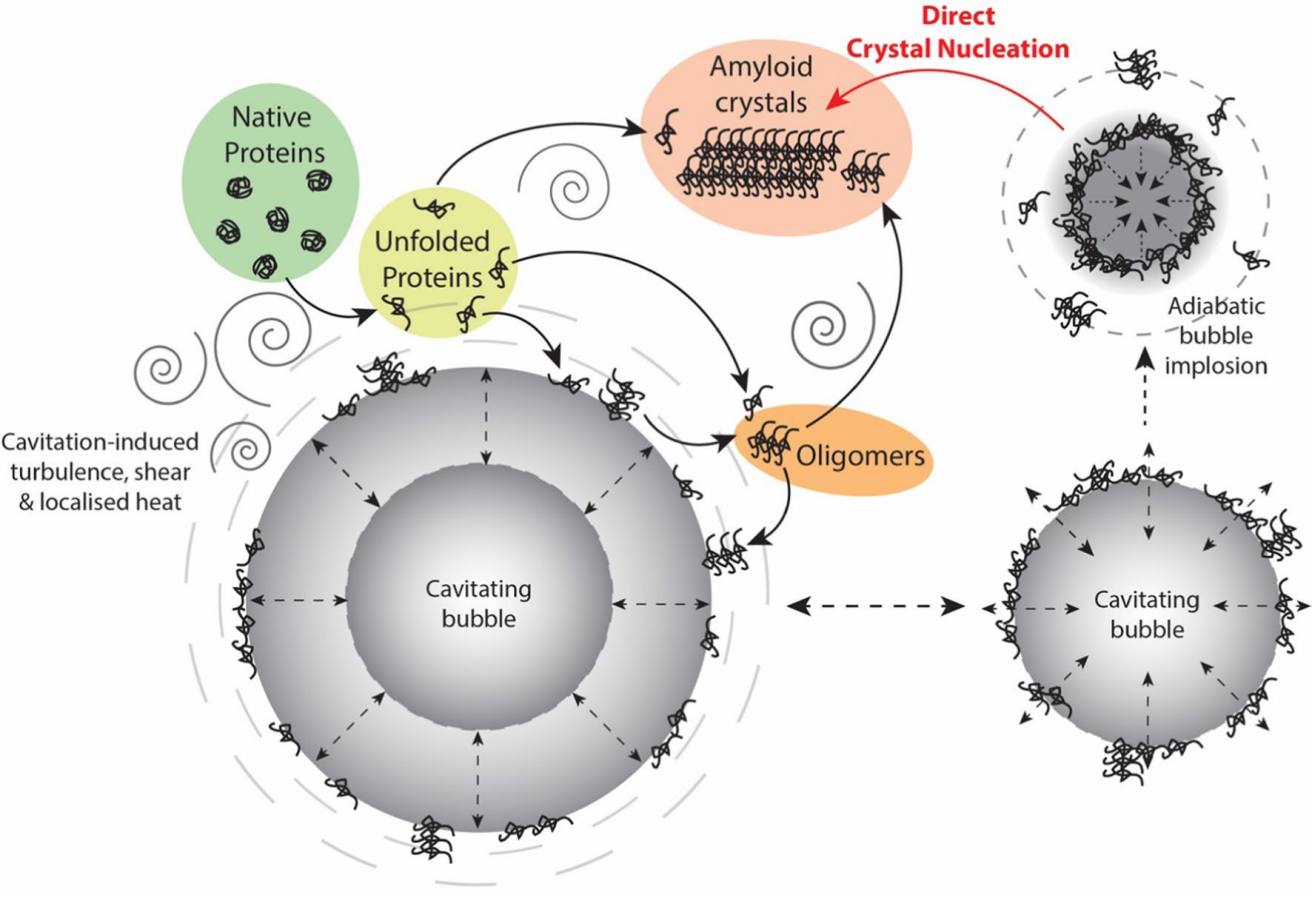
Proposed mechanism for ultrasonic protein aggregation. A schematic illustration of possible mechanistic pathways for amyloid crystal formation by low-frequency ultrasound. Firstly, the localised shear and heat cause protein denaturation, exposing the hydrophobic portions, that enable unfolded proteins to gather at the liquid–bubble interface. Protein–protein interactions occur at the interface to produce oligomers, which can detach from the interface and contribute to the growth of protein crystals. During adiabatic bubble implosion-driven, proteins at the interface of collapsing bubbles can collide with sufficient energy during implosion for catalytic events to surpass the required energy barriers and condense into ordered aggregates as amyloid crystals

Based on consideration of the combined results from this study, it is possible to propose a mechanism for ultrasound-induced formation of amyloid crystals from soluble proteins (β-lg, lysozyme, PPI, native whey protein). Ultrasound can cause partial unfolding and denaturation of proteins (Chandrapala et al. 2012; Villamiel and de Jong 2000). Therefore, we propose that the combination of localised heat (despite maintenance of ambient bulk fluid temperature) and intense shear resulting from ultrasonic cavitation cause sufficient unfolding of native protein molecules (Chandrapala et al. 2012) in the bulk phase and expose the hydrophobic regions present in its core (a visual summary is shown in Fig. 12). Such exposed hydrophobic regions enable intermolecular aggregation (Wang and Roberts 2018). Once unfolded, the increase in hydrophobicity would facilitate the protein molecules to reside at the solution–bubble interface. In turn, unfolded proteins will interact with proteins at the interface, which would act as sites for crystal nucleation to occur. The oscillating acoustic bubbles would thereby serve as a template for the nucleation process, in which β-sheet stacked nuclei are produced and released for subsequent amyloid crystal growth. The cavitation-induced shear would also facilitate the mass transfer of unfolded proteins, to accelerate crystal growth, which the data above suggest is not rate-limiting. Most relevant to the current work, a similar study (Nakajima et al. 2016) discussed earlier proposed that the cavitation bubble on account of it providing physical spherical surfaces acts as a ‘nucleation factory’ for amyloid fibrillation. They also used microbeads to substitute the spherical cavitation bubble surface. However, the microbeads did not produce similar experimental results, as their mathematical modelling had not taken into account the catalytic attributes of cavitation bubble arising from the extremes of local heat and pressure, and high shear due to ultrasonic oscillations. They used pulsed ultrasound for nucleation but did not seek to grow amyloid fibrils using continuous sonication.

A significant observation made in the present work was the production of amyloid crystals in the micron range as opposed to the amyloid fibrils synthesised in the nano-size range seen in previous studies (Dunstan et al. 2009; Jones et al. 2010; Loveday et al. 2010). Until recently, it was believed that amyloid fibrils were the most stable protein aggregates (Hartl et al. 2011; Hartl and Hayer-Hartl 2009), but this information is now updated as newer structural features were observed in amyloid fibrils. Untwisted crystalline tailing parts of amyloid fibrils have since been observed (Adamcik and Mezzenga 2018; Reynolds et al. 2017) that are apparently formed from portions of amyloid fibrils able to traverse a *torsion energy barrier* to acquire a crystalline form. It has, therefore, been theorised that several relative energy minima exist in the amyloid state, and that amyloid crystals are most likely to occupy the ground state (Adamcik and Mezzenga 2018). Thus, if ordered protein aggregates can cross the torsion energy barrier, they can attain an even lower energy state of an amyloid crystal—its thermodynamically most stable form.

**Fig. 13:**
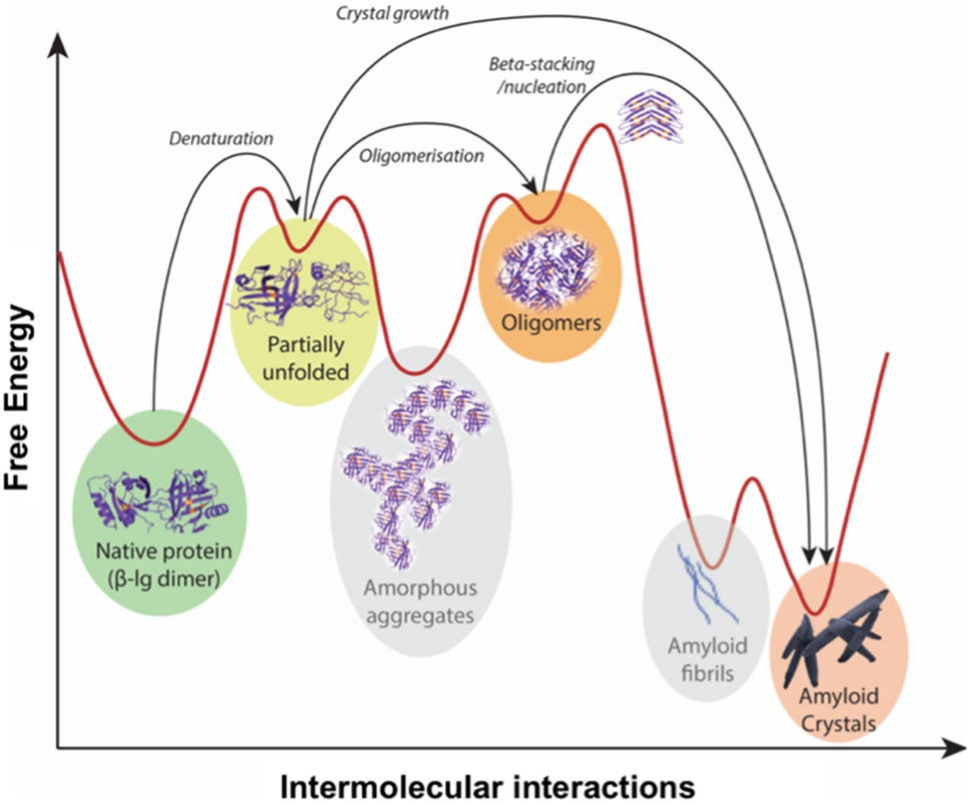
Proposed mechanism of synthesis in the energy landscape (Adamcik and Mezzenga 2018; Balchin et al. 2016) for amyloid crystal synthesis with ultrasound. The protein structures were generated in PyMol (Schrodinger 2010) with data from Protein Data Bank code 1CJ5 (Kuwata et al. 1999)

Surprisingly, fibrils were not observed in the present study. The formation of amyloid crystals in the present study is unusual as most previously documented amyloid structures are either twisted or helical fibrils (Adamcik et al. 2010; Dunstan et al. 2009; Lara et al. 2014; Zhang et al. 2013). As per the energy landscape for protein folding and aggregation (Adamcik and Mezzenga 2018; Reynolds et al. 2017), a considerable energy requirement exists for protein aggregates to overcome the torsion energy barrier and reach the amyloid crystal state, instead of forming amyloid fibrils. The formation of amyloid crystals for longer proteins is expected to be less accessible experimentally due to entropic restrictions (Adamcik and Mezzenga 2018). The cavitation bubbles produced during sonication provide reactive surfaces for catalysis of high-energy reactions, for example the synthesis reaction of sono-assembled nanoparticles composed of phenolic oligomers (Cavalieri et al. 2016). Therefore, we propose that acoustic cavitation catalyses amyloid crystal formation by overcoming entropic restrictions in the restructuring of protein molecules (Fig. 13). The adiabatic implosion of the acoustic bubbles imparts enough energy to the protein aggregates to help cross the untwisting barrier (Adamcik and Mezzenga 2018) and achieve the most stable crystal state of amyloid aggregation (in theory).

To summarise the understanding from this study, it would facilitate to assort information as (i) the inferences drawn from this study, (ii) the contextual background from literature that supports this information, and (iii) the speculative aspects that could be rationally proposed on the basis of theoretical understanding and experimental outcomes but still remain unconfirmed.

(i) The experimental findings show that ultrasound can cause unfolding and partial denaturation of native protein molecules. Sonication can promote formation of protein oligomers that accumulate alongside crystal formation. However, crystals are formed even before measurable amounts (detection limits of SEC, PAGE) of unfolded proteins and oligomers occur in solution. This suggests that amyloid crystals develop very rapidly relative to the process of nucleation. Amyloid crystals form and continue to grow, with no evidence of fibril formation—either as intermediates or end point products.
(ii) It is known from relevant literature that, in order for protein aggregates to attain amyloid crystal state in lieu of amyloid fibril state, the proteins must overcome a torsion energy barrier. It is reasonable to presume that this could happen in a sonochemical system due to acoustic cavitation, possibly the adiabatic bubble implosion providing the energy needed for crossing this threshold. Sonocrystallisation of non-protein molecules has been studied, and it is known that ultrasound induces uniform and faster nucleation that improves crystallization. It is also established that amyloid formation follows crystallization kinetics. Therefore, it can be said that, that even though nucleation seems to be the rate limiting step of amyloid formation, the ease of nucleation induced by ultrasound may be the underlying reason for amyloid crystal formation, intensified by extreme shear.
(iii) Remaining uncertain aspects are the exact site (bubble surface/ bulk solution/ imploding bubble), cause and form of nucleation (interfacial nucleation or adiabatic bubble implosion-driven nucleation). The repeating protein unit of deposition (single or oligomeric units) is also unknown. In terms of the mechanism of deposition, it remains unclear whether or not (and to what extent) crystal growth is accelerated by cavitation/bubble implosion. The relative rates of new crystals vs. crystal growth and what ultimately limits the process is still unknown. It could be that the bubble surface area or the number or concentration of the imploding bubbles is limiting, or rather that the coincidence in time/space of sufficient number of unfolded proteins and imploding bubble is the dominant factor.

### Potential applications of ultrasound-induced amyloid crystal formation

As demonstrated in the present study, ultrasound-induced amyloid crystal formation can be carried out at ambient temperature conditions and in acidic, basic and neutral solution. Additional initiator substances are not required. Such ease of synthesis can have utility in nanofabrication for protein-based nanomaterials. The ordered assembly of proteins or peptides into nanosized amyloid structures under varied conditions is being actively researcher for potential nanobiotechnological applications (Hamley 2007). The current method of amyloid crystal formation presents an opportunity that could be explored for relatively faster and less fastidious formation of nanomaterials with starting materials suited to different synthesis environments (pH, temperature, concentration, ionic solvents). Therefore, ultrasound-induced amyloid crystal formation could be of use in nanobiotechnology (Hamley 2007; Wei et al. 2017) and bioengineering (Jacob et al. 2015).

**Fig. 14:**
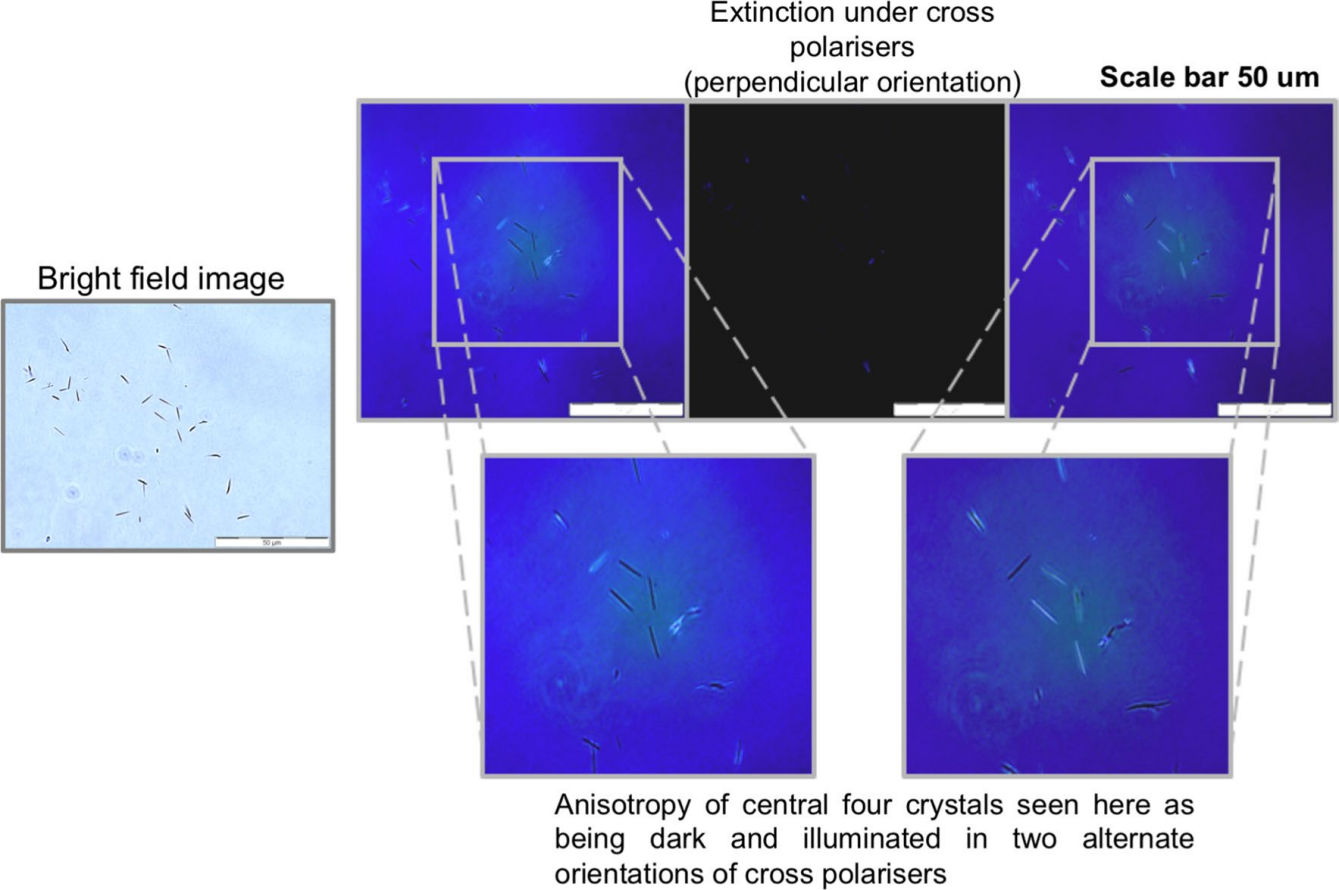
Birefringence exhibited by β-lg amyloid crystals. Amyloid crystals synthesised with low-frequency ultrasound (20 kHz) as observed with polarized optical microscopy. The sample was sonicated at 20 kHz for 60 min at 20 W/cm^3^, 20 ± 1 °C, 7.0 ± 0.4 pH. Scale bars is 50 μm

The β-lg amyloid crystals formed in this study were optically anisotropic and exhibited birefringence under polarised microscopy (Fig. 14). The anisotropy is a result of their large aspect ratios due to enhanced linear growth. The birefringence that was observed for amyloid crystals is consistent with the previous findings for β-lg amyloid fibrils (Bolder et al. 2006; Jung and Mezzenga 2010). As such, there is scope for ultrasound-induced amyloid crystals to be used for biomedical selective imaging applications involving anisotropy, or in biosensors based on their optical activity.

In addition, the present study can potentially be expanded to experimentally support molecular dynamics (MD) simulation studies on proteins to study denaturation, aggregation (Euston et al. 2007) and amyloid formation (Gowda et al. 2021). For example, a MD study of β-lg in food systems (Euston et al. 2007) generated simulations of protein denaturation and amyloid aggregation. The simulations assumed a temperature of 500 K, dimensions of 7 × 7 × 7 nm and conformations were recorded at a timescale of picoseconds. Acoustic cavitation can achieve higher temperatures on that scale of dimensions, within that time range and, therefore, ultrasound synthesis of amyloid crystals could potentially inform the theoretical understanding with useful experimental data.

## Conclusions

Mesoscopic amyloid crystals were produced using low-frequency (20 kHz) ultrasound. This work reports the formation of β-lg amyloid crystals at neutral, acidic and basic pH and ambient bulk temperatures, under a wider and more flexible range of ultrasound operation and solution parameters. These amyloid crystals exhibited crystallization kinetics in growth, autofluorescence, β-stacking shown by ThT fluorescence, increased antiparallel β-sheet structure, and birefringence. The results from this work contribute to a better understanding of the mechanism of formation of amyloid crystals. Amyloid crystals are regarded thermodynamically as the most stable state of protein aggregates but are difficult to synthesise due to existence of energy barriers. The formation of ultrasound-induced crystals proposed here is not specific to β-lg (used as a model protein) and it does not rely on a particular tertiary or quaternary structure of the protein, since other proteins, such as lysozyme, PPI and native whey protein, were shown to form similar amyloid crystals in this study. The growth of amyloid crystals was found to be dependent on sonication time and power. There is scope to study the effect of various ultrasound and solution parameters on fine tuning the synthesis process and producing variably sized structures. Bubble-dependent parameters could be varied to understand the role of bubble implosion in nucleation and mass spectrometric and X-ray methods could be employed to examine the composition and organisation of the crystals and oligomeric intermediates. With potential applications in nanobiotechnology and bioengineering, ultrasound-induced formation of amyloid crystals is an area of active research focus.
